# Unconditional cash transfers for preterm neonates: evidence, policy implications, and next steps for research

**DOI:** 10.1186/s40748-023-00173-1

**Published:** 2024-01-05

**Authors:** Zoe Bouchelle, Timothy D. Nelin, Elizabeth Salazar, Andrea F. Duncan, Margaret G. Parker

**Affiliations:** 1 Department of Pediatrics, Denver Health, Denver, CO USA; 2 PolicyLab, Children’s Hospital of Philadelphia, Philadelphia, PA USA; 3 Guaranteed Income and Health Consortium, Johns Hopkins Bloomberg School of Public Health, Baltimore, MD USA; 4 Department of Pediatrics, Perelman School of Medicine, University of Pennsylvania, Philadelphia, PA USA; 5 Division of Neonatology, Children’s Hospital of Philadelphia, Philadelphia, PA USA; 6 Center of Excellence in Environmental Toxicology, University of Pennsylvania Perelman School of Medicine, Philadelphia, PA USA; 7 Perelman School of Medicine, Leonard Davis Institute of Health Economics, University of Pennsylvania, Philadelphia, PA USA; 8 Department of Pediatrics, UMass Chan School of Medicine, Worcester, MA USA

## Abstract

To address socioeconomic disparities in the health outcomes of preterm infants, we must move beyond describing these disparities and focus on the development and implementation of interventions that disrupt the factors contributing to them. Unconditional cash transfers (UCTs), which provide unrestricted payments to individuals or households, can help mitigate income disparities and improve health outcomes. While UCTs have been utilized for other vulnerable populations, their full potential has yet to be realized for low-income families with preterm infants, who face significant financial strain. In this perspective, we review evidence supporting UCTs as an intervention for children in the U.S. (including those born term and preterm), discuss the potential benefits of recurring UCTs to low-income families of preterm infants, and propose a conceptual model through which UCTs may improve outcomes for preterm infants. We conclude with potential policy levers for implementing UCTs and key unanswered questions for researchers.

## Introduction

To address socioeconomic disparities in the health outcomes of preterm infants, we must move beyond describing these disparities and focus on the development and implementation of interventions that disrupt the factors contributing to them. Unconditional cash transfers (UCTs), which provide unrestricted payments to individuals or households, are a novel upstream intervention that may help alleviate income disparities and improve infant and caregiver health outcomes. While UCTs have been utilized for other vulnerable populations, their use has not yet been fully realized among low-income families with preterm infants.

UCTs may be particularly beneficial for families of preterm infants, particularly those who have low incomes for multiple reasons. First, prolonged hospitalizations and post-discharge care for children with medical complexity (like preterm infants) can be financially burdensome and exacerbate preexisting financial stressors for low-income families [1–4]. Second, preterm infants born in low-income families – who, due to structural racism are disproportionately children of color – also suffer worse health outcomes compared to infants born in higher-income families [5]. Evidence suggests that 50% of health is determined by socioeconomic and environmental factors, while the provision of clinical care influences only 20% [6]. These disparities in health outcomes have not closed with medical advances alone and will require social interventions to narrow and ultimately eliminate. Finally, on a societal level, the costs of prematurity-associated morbidity are significant (estimated at over $26 billion annually) [7].

Thus, UCTs are an upstream social intervention with the potential to improve the health and well-being of preterm infants and their families, advance health equity, and reduce the costs of prematurity-associated morbidity. In this perspective, we review evidence supporting unconditional cash transfers as an intervention for children in the U.S. (including those born term and preterm), discuss the potential benefits of UCTs to low-income families of preterm infants, and propose a conceptual model through which UCTs may improve outcomes for preterm infants. We conclude with potential policy levers for implementing UCTs and key unanswered questions for researchers.

## Cash transfer background

Cash transfers are payments made directly to individuals or households without restrictions on how the money is spent. They can be delivered conditionally or unconditionally.

Conditional cash transfers (CCTs) are made to individuals or households on the condition that they fulfill certain obligations. For example, households may receive conditional cash transfers for taking their children to preventative health visits, receiving vaccinations, or breastfeeding. One benefit of CCTs is their ability to target specific health-promoting behavior change. However, a drawback of CCTs is that they place administrative burdens to confirm that recipients have met specified obligations to receive the CCTs. These administrative burdens affect those who oversee CCT programs, which require the establishment of costly and time-intensive systems to track compliance with requirements for CCT disbursal. In addition, navigating these systems can be time-intensive and stressful for families, ultimately preventing receipt of cash among households that may need it most.

In contrast, UCTs are made to individuals or households without any obligations or requirements attached. While UCTs do not target specific health-promoting behaviors, they may reduce administrative burdens associated with tracking compliance for those disbursing UCTs and families eligible for them. While we focus specifically on UCTs in this perspective, we acknowledge the growing body of work on the use of CCTs to incentivize caregiving and other specific health-promoting behaviors in the neonatal population [13].

## Cash transfers versus in-kind support

Both UCTs and CCTs are distinct from other social welfare programs that provide assistance through specific goods or services (e.g., food stamps, housing, or transportation vouchers). UCTs and CCTs are more flexible, allowing recipients to use the money to meet their most pressing needs, whether it be paying for food, rent, utilities, transportation, medical care, or addressing other urgent expenses. This offers a unique benefit, given the ability of a single intervention to address a myriad of social drivers of health.

## Evidence supporting unconditional cash transfers as a health intervention

A wealth of evidence supports the negative impacts of poverty on children, both during childhood and across their lifetime [14, 15]. Preterm infants are at risk for numerous adverse health outcomes including neurodevelopmental impairment, bronchopulmonary dysplasia, prolonged hospital stays, and increased healthcare utilization across the lifespan. A growing body of research suggests that preterm infants born with in low-income families – who, due to structural racism are disproportionately children of color – also suffer worse health outcomes compared to infants born in higher-income families [5]. In the U.S., Black infants are 50% more likely to be born preterm and three times more likely to be born before 28 weeks’ gestation [8–10]. Black preterm infants experience higher mortality rates and higher readmission rates compared to White preterm infants [11, 12]. UCTs represent a promising intervention to address upstream drivers of these economic and health disparities.

A growing body of evidence supports the impacts of UCTs on child health, however much of the evidence on the impacts of UCT programs on children derives from low- and middle-income nations. For example, a recent Cochrane Review examining UCTs in low- and middle-income countries suggests that they may improve several health outcomes including reducing illness, food insecurity and lack of dietary diversity, as well as improving school adherence and reducing household poverty [16]. While these results are promising, it is important to consider that the applicability of findings from low- and middle-income countries to the U.S. context may be limited, due partly, to the substantial differences in the social, economic, political, and healthcare landscape between the U.S. and low- and middle-income countries.

At present, in the U.S. there are few examples of “purely unconditional” cash transfer programs. Of the many U.S. programs and policies that disburse cash, most are conditional on certain requirements. For example, the Earned Income Tax Credit (EITC) is conditional on having earned income, filing taxes, and claiming the credit, so that it does not benefit families with no income, those eligible for the credit who do not file taxes, or those eligible for the credit who file taxes but fail to claim the credit. In addition, Temporary Assistance for Needy Families is conditional on several requirements which vary by state, like active engagement in job training [17, 18].

In a recent scoping review by Shah et al., the authors review early experimental evidence on cash transfers in the U.S. with a focus on the negative income tax (NIT) experiments in the 1970s [19]. The NIT experiments tested the effects of guaranteed income through randomized trials among low-income households. Evidence from all four United States NIT experiments on children’s health and education suggest the transfers improved children’s school attendance and performance, decreased the incidence of low birth weight, and increased the consumption of nutritious foods [19].

The authors also review quasi-experimental evidence of U.S. unconditional and “nearly unconditional” cash transfer programs and policies on child health [19]. These include data from the Mother’s Pension Program in the early 1900s (a precursor to Aid to Dependent Children), the Alaska oil dividend payments (which began in the 1980s and continue today), the casino dividends paid to East Cherokee families in North Carolina (which began in the 1990s and continue to the present day), federal and state EITC expansions, and the recent (albeit temporary) 2021 Child Tax Credit (CTC) expansion [19]. Taken together, the evidence suggests that these U.S.-based unconditional and “nearly unconditional” cash transfer programs and policies have been associated with positive impacts on several markers of child health including higher birth weights, lower rates of childhood obesity, increased educational attainment, and increased food security [19, 20].

In the scoping review, Shah et al. also summarize nine contemporary, recurring UCT *experiments* directly targeting families with children in the U.S [19]. Of the nine reviewed, the *Baby’s First Years* study is the only newborn-targeted, recurring UCT experiment with evaluation results published in academic journals [19]. In the *Baby’s First Years* trial, 1000 mothers of healthy, term infants in four U.S. cities were randomized to receive either $333 or $20 monthly UCTs for the first four years of their children’s lives [21]. Early evidence from the study has found that, compared to those in the $20/month arm, those in the $333/month arm increased spending on child-specific goods and mothers’ early-learning activities with their infants [22] and impacts on infant brain activity [23].

In the preterm population, to our knowledge, only one randomized controlled trial has explored the impacts of UCTs for families of preterm infants [24]. In the trial, 46 mother-infant dyads were randomized to receive $200 per week for a maximum of three weeks versus no cash [24]. Results suggest that mothers in the treatment arm were more likely to visit their children in the NICU and provide skin-to-skin care and breastmilk while their infant was in the NICU [24].

Larger studies in the preterm population are now underway, including an NIH-funded randomized trial (1R01HD109293-01), which will enroll 420 low-income mothers with infants 25–33 weeks’ gestation in four level-three safety-net NICUs in Massachusetts and Georgia. Mothers will be randomized to receive an unconditional cash transfer of $160 per hospital week versus no cash. Investigators will study the impact on NICU caregiving behaviors, including breastfeeding and skin-to-skin care, potential mechanisms of action, and maternal perspectives of financial transfers (M.G. Parker, personal communication, July 16, 2023).

## Potential mechanisms of action

Despite the historical evidence and contemporary research on the impact of UCTs, the mechanisms through which UCTs may yield improvements in child health outcomes have not yet been fully elucidated. Few models have been published detailing how UCTs may improve child health and to our knowledge, no model has been put forth suggesting mechanisms in the preterm population [14, 25].

**Fig. 1:**
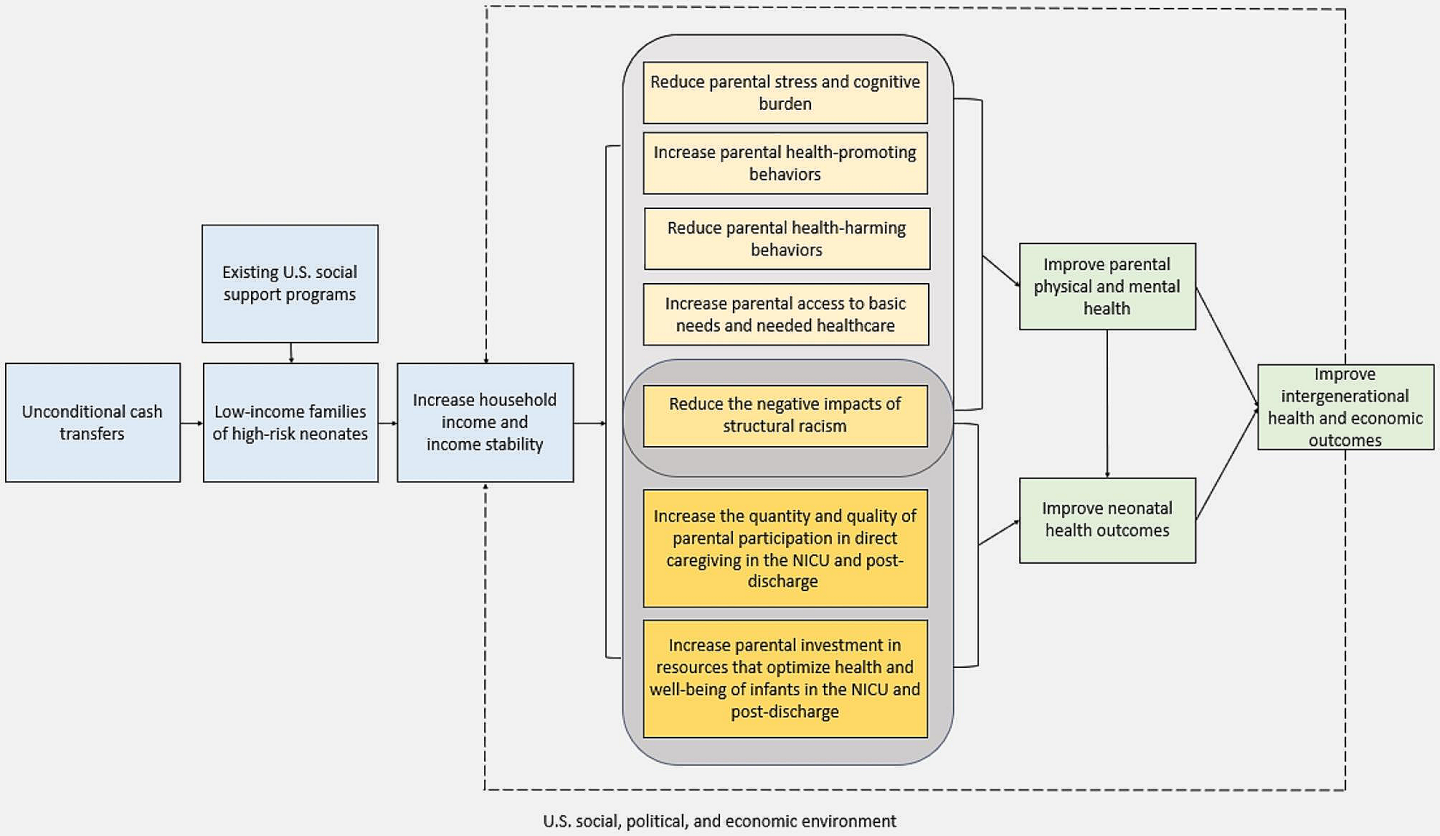
Conceptual model highlighting potential pathways for unconditional cash transfers to improve perinatal health outcomes

To guide future research, we propose a conceptual model in which UCTs have the potential to improve the health and well-being of preterm infants and their caregivers through two main pathways: increased household income and increased income stability (Fig. 1). In our model, household income refers to the annual income received by a household. In contrast, income stability refers to variability in income over time, which may be smoothed with periodic cash transfers. For example, a single lump sum cash transfer of $6,000 annually may increase a household’s annual income, but may decrease the household’s income stability. In contrast, a $500 monthly cash transfer totaling $6,000 over the course of one year may both increase a household’s annual income and increase the household’s income stability month-to-month.

The model highlights 4 key mediators through which increased income and income stability may improve parental health and 3 key mediators through which increased income and income stability may improve neonatal health. We include both infant and parental health as outcomes in the model given the influence of parental health on child health [26, 27] While these mediators are represented together in the model for simplicity, they are interrelated in myriad ways. For example, reduced parental stress may lead to increased parental health-promoting behaviors; but increased parental health-promoting behaviors may also contribute to reduced parental stress. Further, reducing the negative impacts of structural racism has a unique relationship to each mediator identified. Because of the challenges of representing these relationships coherently with individual vectors in the model, we represent them a simplified way while acknowledging their interrelatedness. More research is needed to disentangle the relationships between these mediators.

In the model, increased income and income instability may improve parental mental and physical health via reducing parental stress and cognitive burden, increasing parental health-promoting behaviors, reducing parental health-harming behaviors, increasing parental access to basic needs and needed healthcare, and reducing the negative impacts of structural racism on parental health. Improving parental physical and mental health may lead to improvement in neonatal health, each of which may lead to a positive feedback loop through improving intergenerational health and economic outcomes. Improved parental and mental health may contribute to improved household economic outcomes in the short- and long-term by reducing foregone or missed work due to parental or child illness or increasing parents’ ability to engage in the labor market.

In the model, increased income and income stability may improve neonatal health outcomes by increasing the quantity and quality of parental participation in direct caregiving in the NICU and post-discharge, increasing parental investment in resources that optimize health and well-being of infants in the NICU and post-discharge, and reducing the negative impacts of structural racism on neonatal health. In the NICU, increasing the quality and quantity of parental participation may translate to an increase in the time spent on activities like skin-to-skin care and breastfeeding. After discharge, this may translate to increases in the amount and quality of time engaging in activities like developmentally stimulating play, tummy time, and reading books. UCTs may also help mitigate inequities in parental leave for low-income families (i.e., with more financial support, parents may not need to return to work as quickly) and allow them to engage more frequently in the care for their infants, which has been associated with improved breastfeeding and neurodevelopmental outcomes [28–31].

In addition, increasing parental investment in resources that optimize the health and well-being of infants in the NICU translate to investments in lactation support, such as access to breast pumps, or other items needed to engage in NICU care. After discharge, these resource investments may include enrollment in high-quality childcare, attendance at medical or subspecialty appointments, improved ability to cover the costs of their infant’s ongoing medical needs, and increased ability to engage in other support services (like physical therapy, occupational therapy, speech therapy, Early Intervention). In addition, caregivers may be better able to afford developmentally stimulating items and activities for the home [32], more nutritious food, and higher quality housing.

## Policy options to implement unconditional cash transfers

Several potential policy options exist to implement UCTs in the U.S. for low-income families with children, some of which would benefit low-income children more broadly and some which may specifically target preterm infants.

One policy avenue to implement UCTs to benefit low-income children more broadly is through a revived, expanded Child Tax Credit, akin to the temporary reforms instituted in 2021 during the COVID-19 pandemic. The reforms increased the size of the credit, expanded eligibility for the full credit to families with low or no incomes, and allow families to receive a portion of the credit in advance monthly cash payments. There is precedent for these types of monthly payments to families with children in other high-income countries through tax credits and child allowance programs [33, 34].

Opportunities also exist beyond the federal level as many states have implemented Child Tax Credits [35] and Earned Income Tax Credits to supplement the federal programs [36]. While state-level credits are smaller than the federal credit and often delivered yearly, state legislatures may be more agile than the federal government to implement expansions and reforms to the eligibility, amount, and frequency of the credits on a state-by-state basis [37]. Numerous examples also exist on a local level, including programs such as the Abundant Birth Project in San Francisco which was the first pregnancy income supplement program in the United States. The Abundant Birth Project is also a notable example of a public-private partnership, which may serve as a model for hospitals and health systems to directly support cash transfer programs aimed at improving health.

Medicaid may be an avenue to deliver UCTs that target the preterm population if UCTs were permitted through Sect. 1115 demonstration waivers, which allow for innovation in the Medicaid program. If evidence suggests cost-effectiveness (with short-term UCTs to a preterm population having positive effects on healthcare utilization and costs of care), Medicaid policymakers may be willing to pilot programs to deliver cash support to families.

Federal Supplemental Security Income (SSI) payments represent another policy lever that targets preterm infants. Currently, low birthweight infants, infants with birthweights below thresholds for their gestational age, and infants with growth failure combined with a developmental delay between birth and age three qualify for monthly SSI payments [38]. However, SSI payments often take months to kick in and, while the average SSI monthly payment is over $600, payments are capped at $30 per month while infants are hospitalized despite the fact that the prolonged separation during the NICU hospitalization is a time of considerable financial strain. There are opportunities to test policy changes that may more effectively deliver SSI cash benefits to families of preterm infants including (1) expansions of eligibility, (2) modifications of approval processes so that families receive the payments within one month of a qualifying infant’s birth, and (3) elimination of the $30 per month cap during the initial NICU stay so that early payments equal the amount paid at the time of discharge [39].

## Potential drawbacks of UCTs

UCTs hold the potential to act as an instrument to decrease disparities in health outcomes of preterm infants, financial outcomes of their caregivers, and to advance health equity. However, it is important to note several consequences may exist.

First, while providing supplemental income, UCTs may result in a “cliff effect”, in which families who receive the UCTs, may become ineligible for vital federal and state assistance programs such as Medicaid, WIC, SSI, or housing benefits due to exceeding income thresholds [40]. For this reason, UCT programs must be designed and implemented thoughtfully. Recipients would likely most benefit from UCTs being implemented as a supplement to, instead of a replacement for, existing social programs. In addition, recipients would likely benefit from having income received through UCT programs exempted from calculations determining their eligibility for existing social programs to avoid the “cliff effect”, which may inadvertently make recipients financially worse off.

Second, while large studies such as Baby’s First Years did not detect negative impacts on employment, there is the potential for workforce participation reduction depending on the monetary value of the UCT [41]. Any detected reductions in work participation among parents of newborns, however, must be interpreted thoughtfully, given the well-described benefits of parental time home with infants during the early months of their lives. Lastly, due to wide variations in the cost of living throughout the United States, UCTs delivered as a uniform value regardless of regional cost of living differences may result in a relative disadvantage for households residing in higher-cost areas compared to those in lower-cost areas.

## Next steps for research

The potential impact of UCTs on the health and well-being of preterm neonates has received little investigation. We highlight eight important areas for future study and pose key unanswered questions to guide future investigation among perinatal researchers (Table 1). The eight areas of study include the (1) optimal UCT “dose”, (2) optimal UCT frequency, (3) optimal UCT timing, (4) impacts of UCTs on health outcomes, (5) mechanisms of action, (6) impacts on healthcare utilization, (7) cost-effectiveness, and (8) unintended negative consequences of delivering UCTs.

**Table 1:** Next steps for research on unconditional cash transfers for preterm infants and their caregivers

**Area of Study and Key Unanswered Question **	**Comments **
**Dose:** What is the optimal “dose”, or monetary amount of a UCT, to produce meaningful outcomes for preterm neonates?	More research is needed on the optimal UCT “dose” for preterm neonates. Researchers must consider that the “dose” needed may vary with the outcome under study (i.e. the “dose” needed to improve the frequency of parental visitation during a NICU stay may be smaller than the “dose” needed to impact infant mortality).
**Frequency:** What is the optimal UCT frequency to produce meaningful outcomes for preterm neonates?	More research is needed on the optimal UCT frequency for preterm neonates. Researchers must consider that UCTs can be delivered as large, infrequent sums (like the yearly delivery of the EITC), or in smaller, more frequent sums (like the delivery of monthly payments with the 2021 CTC expansion).
**Timing:** What is the optimal UCT timing to produce meaningful outcomes for preterm neonates?	Ideally, UCTs be timed to begin pre-conception or during pregnancy as an intervention to prevent preterm birth. However, in the absence of these policies in the prenatal period, researchers should consider UCTs for preterm neonates. More data are needed on the timing of cash interventions after birth - including whether they are more effective during the NICU stay, after the NICU stay, or during both time points.
**Health Outcomes:** Do UCTs improve neonatal health outcomes?	Key infant health outcomes may include infant mortality, morbidity, and neurodevelopment (both short- and long-term). Beyond describing impacts on health outcomes in the aggregate, more data are needed on health impacts stratified by race and ethnicity as a measure of health equity, as well as qualitative data on the lived experiences of families.
**Mechanisms of Action:** What are the mechanisms through which UCTs may impact neonatal health outcomes?	Mediators along the causal pathways may include impacts on household income and income stability, caregiver stress and cognitive burden, caregiver health and well-being, the quantity and quality of caregiver time investments in the care of their infant, and caregiver access to resources to optimize the health and well-being of their infants.
**Healthcare Utilization: **Do UCTs impact healthcare utilization among preterm neonates?	This may include measures of the impact of UCTs on primary care and neonatal follow-up program attendance, engagement in physical, occupational, and speech therapy, enrollment in Early Intervention, and acute care utilization.
**Cost-Effectiveness: **Are UCTs delivered to families of preterm neonates cost-effective?	More data are needed on the extent to which early periodic investments may reduce costs in the long term. Reductions in costs for preterm neonates may be related to costs of the initial NICU stay, costs of subsequent acute care and readmissions, costs associated with prematurity- related morbidity including specialized medical care and special education services, and lost household and labor market productivity (both for the parent and the child later in adulthood) associated with the child’s morbidities.
**Unintended Negative Consequences:** What are the potential unintended consequences related to the delivery of UCTs to preterm neonates?	Some families may lose government benefits due to the increased income from UCTs. This is often called the “cliff effect,” in which families may experience a small increase in income that results in them exceeding income limits for several government benefits, even by a small amount. While many pilots can pursue waivers to exempt the income from being taxable or from impacting government benefits, some government benefits are highly likely to be affected (like Supplemental Security Income). It is also possible that UCT policies may be implemented to replace (as opposed to complement) current social safety net programs, which may have unintended consequences and could result in a net welfare loss to families should the UCT received have a lower value than the social safety net programs it replaced. Families may also have reductions in other earned income through deferred work. Measurements of impacts on earned income will require a nuanced approach and careful selection and interpretation of outcome measures. UCTs may allow caregivers to spend more time with their infant before returning to work, which could result in lower earned income and may not reflect the positive impact of the cash transfer.

## Conclusion

UCTs are an upstream social intervention with tremendous potential to improve the health and well-being of preterm infants and their families, advance health equity, reduce costly acute care utilization, and reduce long-term health spending through prevention. More policy-oriented research is urgently needed to examine the “dose,” frequency, and timing effects of UCT interventions, the impact of UCTs on health outcomes of preterm neonates and their families, the mechanisms through which potential health impacts may be mediated, and the cost-effectiveness of UCT interventions.

## Data Availability

Not applicable.
